# A single bacterium restores the microbiome dysbiosis to protect bones from destruction in a rat model of rheumatoid arthritis

**DOI:** 10.1186/s40168-019-0719-1

**Published:** 2019-07-17

**Authors:** Hudan Pan, Ruijin Guo, Yanmei Ju, Qi Wang, Jie Zhu, Ying Xie, Yanfang Zheng, Ting Li, Zhongqiu Liu, Linlin Lu, Fei Li, Bin Tong, Liang Xiao, Xun Xu, Elaine Lai-Han Leung, Runze Li, Huanming Yang, Jian Wang, Hua Zhou, Huijue Jia, Liang Liu

**Affiliations:** 1 State Key Laboratory of Quality Research in Chinese Medicine/Macau Institute for Applied Research in Medicine and Health, Macao University of Science and Technology, Macao, China; 2 BGI-Shenzhen, Shenzhen, 518083 China; 3 China National Genebank, BGI-Shenzhen, Shenzhen, 518120 China; 4 International Institute for Translational Research of Traditional Chinese Medicine of Guangzhou University of Chinese Medicine, Guangzhou, 510006 Guangdong China; 5 Fujian University of Traditional Chinese Medicine, No.1, Qiuyang Road, Minhoushangjie, Fuzhou, 350122 Fujian China; 6 BGI Education Center, University of Chinese Academy of Sciences, Shenzhen, 518083 China; 7 Shenzhen Engineering Laboratory of Detection and Intervention of human intestinal microbiome, BGI-Shenzhen, Shenzhen, 518083 China; 8 James D. Watson Institute of Genome Sciences, Hangzhou, 310058 China

## Abstract

**Background:**

Early treatment is key for optimizing the therapeutic success of drugs, and the current initiating treatment that blocks the progression of bone destruction during the pre-arthritic stages remains unsatisfactory. The microbial disorder in rheumatoid arthritis (RA) patients is significantly reversed with effective treatment. Modulating aberrant gut microbiomes into a healthy state is a potential therapeutic approach for preventing bone damage.

**Results:**

By using metagenomic shotgun sequencing and a metagenome-wide association study, we assessed the effect of *Lactobacillus casei* (*L. casei*) on the induction of arthritis as well as on the associated gut microbiota and immune disorders in adjuvant-induced arthritis (AIA) rats. Treatment of AIA rats with *L. casei* inhibited joint swelling, lowered arthritis scores, and prevented bone destruction. Along with the relief of arthritis symptoms, dysbiosis in the microbiome of arthritic rats was significantly reduced after *L. casei* intervention. The relative abundance of AIA-decreased *Lactobacillus* strains, including *Lactobacillus hominis*, *Lactobacillus reuteri*, and *Lactobacillus vaginalis*, were restored to normal and *Lactobacillus acidophilus* was upregulated by the administration of *L. casei* to the AIA rats. Moreover, *L. casei* downregulated the expression of pro-inflammatory cytokines, which are closely linked to the effect of the *L. casei* treatment-associated microbes*.* Functionally, the maintenance of the redox balance of oxidative stress was involved in the improvement in the *L. casei*-treated AIA rats.

**Conclusion:**

A single bacterium, *L. casei* (ATCC334), was able to significantly suppress the induction of AIA and protect bones from destruction in AIA rats by restoring the microbiome dysbiosis in the gut, indicating that using probiotics may be a promising strategy for treating RA, especially in the early stage of the disease.

**Electronic supplementary material:**

The online version of this article (10.1186/s40168-019-0719-1) contains supplementary material, which is available to authorized users.

## Introduction

Rheumatoid arthritis (RA) is a common systemic autoimmune disease associated with bone destruction [1]. Early diagnosis and treatment play pivotal roles in optimizing the therapeutic success of treatment with drugs, particularly for RA patients with high disease activity, presence of autoantibodies, and early joint damage [2]. Evidence is accumulating that the first few months after the onset of symptoms represent a pathologically distinct phase of the disease, offering a therapeutic window of opportunity to possibly switch off disease progression permanently [3]. However, robust diagnostic biomarkers with high sensitivity in the early stage are still lacking, and the current initiating treatment that blocks the progression of bone erosion during the pre-arthritic stages remains unsatisfactory [4].

A relationship between the gut microbiota and RA has been suspected for many years, and a number of epidemiological and clinical reports have implicated an array of microorganisms in RA pathogenesis. However, causation has not yet been established [5], in part because of the complex interactions between the microbiota and genetic factors such as major histocompatibility complex (MHC) molecules which may affect and modulate the composition of microbial communities [6]. Furthermore, RA-enriched gut microbial genes may act as potential molecular mimics of RA-associated antigens [7, 8].

We recently reported on the first catalog of the gut microbiota of Sprague-Dawley (SD) rats established by shotgun metagenomic sequencing [9]. Profiling of this catalog showed that the gut microbiome of rats compared to mice is slightly more similar to that of humans [9], and at the functional level, 97% of the pathways are shared suggesting that rat at the functional level to a certain extent may serve as a model for humans. The microbial perturbation in RA patients was demonstrated to be partially reversed with effective treatment in our previous study [7], indicating that modulation of the gut microbiome is closely related to the effectiveness of treatment. Several clinical studies have also shown that the use of probiotics might contribute to RA remission when combined with disease-modifying anti-rheumatic drugs (DMARDs) [10]. However, the causation of changes and the dynamic changes in the gut microbiome throughout the progression of arthritis are unclear, and whether a single bacterium used as a probiotic is able to prevent or ameliorate the early stage of disease remains to be proven. *Lactobacillus casei* ATCC334 (*L. casei*) is one of the most-widely used probiotics. Mice fed with *L. casei* could significantly decrease the expression of TLR2 and TNF-α, which were closely related to the pathogenesis of RA [11]. In the present study, we used a well-established RA model, adjuvant-induced arthritis (AIA), in SD rats, which is pathologically similar to human RA [12] to investigate the potential of administration of the probiotics *L. casei* for treatment of RA. We observed that administration of *L. casei* elicited profound changes in the composition of microbial species in the gut along with the changes in clinical symptoms during the processes of arthritis induction and progression.

## Methods

### Animals

SD rats weighing approximately 60 g were maintained under standard laboratory conditions at 25 °C, with a 12 h/12 h light/dark cycle and 55% humidity and fed feed and water ad libitum. The four groups with seven rats in each cage were all fed the regular (low-fat) chow. The rats were allowed 14 days to adapt to the laboratory environment before experiments were initiated. Adjuvant arthritis was induced according to the method described previously [13]. The AIA rats were randomly divided into three groups, each of seven rats, the AIA model (vehicle-treated with 0.3% sodium carboxymethyl cellulose (CMC-Na)), the *L. casei-*treated (2 × 10^8^ colony-forming units (CFU)/day) group, and the methotrexate (MTX)-treated (7.6 mg/kg/week) group. In addition, normal rats were used in the experiment for comparative purposes. The clinical symptoms of arthritis were evaluated through increases in hind paw volume and the arthritis score by two independent observers. Blood samples were collected from the abdominal aorta of anesthetized rats on day 30 of the experiment. Then, the rats were sacrificed by cervical dislocation. The erythrocyte sedimentation rate (ESR) was determined by a modified method based on the method selected by the International Council for Standardization in Haematology (ICSH). The levels of IL-1β, TNF-α, IL-17, and IL-6 in serum were determined using commercially available enzyme-linked immunosorbent assay (ELISA) kits according to the manufacturer's instructions (R & D Systems, USA).

### Metagenomic sequencing and annotation

Single-end metagenomic sequencing was performed on the BGISEQ-500 platform as described previously [14]. Briefly, we constructed one DNA single-end (SE) library with a read length of 100 bp. Low-quality reads and rat reads (categorized according to an alignment with NCBI accession no. NC_005100) were removed. On average, 7.24 Gb of high-quality non-host sequences were obtained per sample (Additional file 15: Table S1). The relative abundances of species determined by the metagenomic sequencing were computed using the MetaPhlAn2 profiling software with default parameters [15] (Additional file 16: Table S2). Reads were mapped to the gene catalog of the SD rat gut metagenome [9] to generate the Kyoto Encyclopedia of Genes and Genomes (KEGG) ortholog and module profiles.

### Health and disease planes

Health and disease planes were constructed as previously described [16]. In brief, principal coordinates analysis (PCoA) of the Bray-Curtis distance was performed at the species level, and then samples in the model or normal group were fitted to a two-dimensional plane at each time point using the least-squares method on the first three principal coordinates. The Euclidean distances from samples to these planes at each time point were calculated.

### Selection of arthritis-correlated microbes

The relative abundance of the microbial species at the five TPs were used to regress to the arthritic scores. (i) Microbial species presenting in less than 5% of rats were removed. (ii) We applied log-transform to the profile of microbial species and subsequently normalized it by z-score. (iii) Five-fold cross-validation with ten repeats was performed on an elastic-net regularized linear regression model (R 3.5.1, Glmnet2.0-16 package) using the normalized profile of microbial species against the arthritic scores. Totally, 50 regression models were obtained and we extracted arthritis-correlated species by two steps: (i) removed models in which the number of nonzero *β* is less than five and (ii) selected species which is presented in more than 25 models.

### Fold change analysis

The relative abundances of the selected arthritis-correlated species in the model, MTX-treated, and *L. casei*-treated groups were normalized by those in the normal group at each TPs and then were calculated in the log_10_ fold change (FC) compared to TP1. The change was defined as significant if a species experienced consistent > 16 FC (log_10_FC values are ± 1.20) in over 80% of the rats.

### Correlation analysis

Semi-partial Spearman correlation tests (R 3.5.1, ppcor package 1.1) were used to calculate the correlation coefficients between the relative abundance of arthritis-correlated microbes and the distances to HP or DP adjusted for weight and group, also between the relative abundance of identified microbes and the serum cytokines measured by commercial ELISA kit, as previously described [17], adjusted for arthritic scores, weight, and group, after removing the outliers.

### KEGG analysis

Putative amino acid sequences were translated from the gene catalogs and aligned against the proteins or domains in the KEGG databases (release 79.0, with animal and plant genes removed) using BLASTP (v2.2.24, default parameter, except –e 0.01 –b 100 –k 1 –F T –m 8). Differentially enriched KO modules were identified according to their W statistic, from Wilcoxon rank-sum test which was performed on all the KOs at day 30.

## Results

### *L. casei* significantly alleviates experimental arthritis

**Fig. 1:**
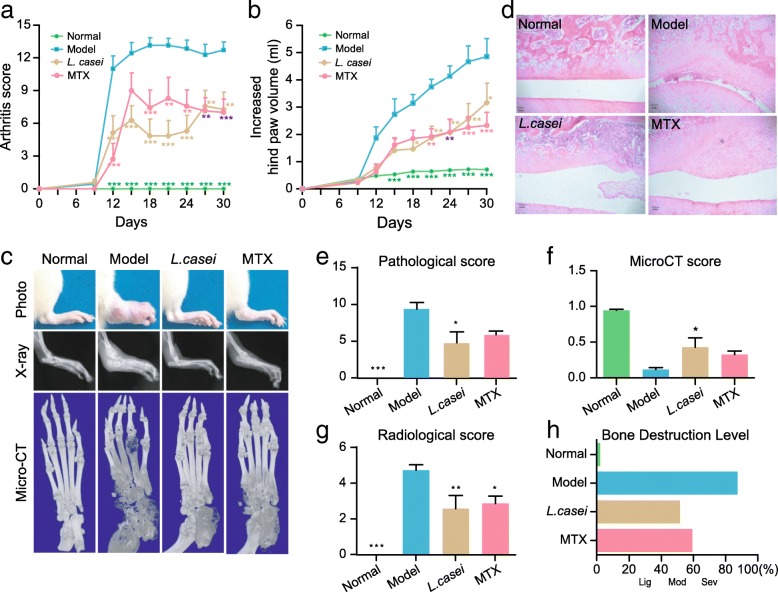
*L. casei* alleviates the adjuvant-induced arthritis of rats. Effects of *L. casei* on arthritis score (**a**) and increased hind paw volume (**b**) are shown (*n* = 7 for each group). Data in **a**, **b** are shown as mean ± s.e.m. Differences between groups are analyzed by two-way ANOVA (*P < 0.05, **P < 0.01, ***P < 0.001 VS model). The photographs, X-ray, and micro-CT images of ankles are shown in **c**. Representative images of pathological sections of knees in rats in different groups are shown in **d**. The pathological improvements are assessed by pathological score **e**. Radiological score and micro-CT score are evaluated using the micro-CT image and micro-CT analyzer, respectively (**f**, **g**). Data in **e**, **f**, and **g** are shown as mean ± s.e.m. Differences among groups are analyzed by one-way ANOVA. (*P < 0.05, **P < 0.01, ***P < 0.001 VS model). The integral assessments of the bone destruction levels are shown in (**h**). Data are shown as mean and classified into several levels. 0–0.2: normal; 0.2–0.4: light (Lig); 0.4–0.6: moderate (Mod); 0.6–0.8: severe (Sev); 0.8 and above: very severe. Normal, normal control; model, disease control; MTX, methotrexate

SD rats were orally gavaged with or without the *L. casei* strain ATCC334 and subsequently challenged with complete Freund’s adjuvant (CFA) on the same day (day 0) [13]. The animals were orally gavaged with 2 × 10^8^ CFU every day from day 0 to day 30. As the culture medium for *L. casei* contains lactic acid and other metabolites, we washed off the medium, re-suspended the bacteria in CMC-Na, and then fed the live bacteria to the SD rats within half an hour. The activity of *L. casei* in CMC-Na was similar to that in MRS broth in such a short time (Additional file 1: Figure S1, Additional file 2: Figure S2). MTX, a widely used DMARD [18], was used as a comparative control. After a 30-day intervention, the rats treated with *L. casei* or MTX showed less aggravated symptoms, as assessed by the arthritis scores and hind paw volumes (Fig. 1a, b), and rats treated with a combined therapy of *L. casei* and MTX revealed an even better improvement than those with MTX or *L. casei* monotherapy (Additional file 3: Figure S3). Moreover, *L. casei* exerted its anti-arthritic effect without migrating to the places beside the gut as *L. casei* is facultative anaerobic bacteria and no bacteremia was detected in the *L. casei*-treated animals.

Bone and cartilage destruction, which often induce partial or permanent disability in poly-joints, are the major characteristics of pathological alterations in RA patients. Here, we used micro-computed tomography (micro-CT) to evaluate the effect of *L. casei* on bone remodeling. The bone erosion was diminished in the ankles of the *L. casei*-treated rats compared to the vehicle-treated animals (Fig. 1c). Consistent with the micro-CT results, histological sections of the knees showed similar improvements after the intervention (Fig. 1d). In general, the pathological scores of the knees and the radiological scores of the ankles were significantly improved in the *L. casei*-treated animals compared to the vehicle-treated animals (Fig. 1e, g). The micro-CT score, calculated using the trabecular bone mineral density (BMD), bone volume rate (BV/TV), trabecular number (Tb.N), porosity percent (Po × total), and tissue mineral density (TMD), suggested severe osteoporosis in the vehicle-treated animals (Additional file 4: Figure S4). Compared with the vehicle-treated rats, the rats treated with *L. casei* exhibited reduced bone erosion with a higher micro-CT score (Fig. 1f). From the three bone destruction evaluation scores, we created a classification scale that equally integrated the scores to more precisely assess the degree of damage. Using this classification scale, the *L. casei*-treated animals showed a significant change in the arthritis level from severe to moderate (Fig. 1h). Moreover, the metabolites produced by *L. casei* were also used separately in the AIA rats, and results were shown with no significant improvement (Additional file 3: Figure S3). These data indicate that administration of *L. casei* was able to alleviate experimental arthritis and protect bones from erosion.

### *L. casei* prevents gut dysbiosis caused by the induction of arthritis

We next examined how the gut microbiota changed during the process of arthritis induction and how the administration of *L. casei* affected these changes. It is important to note that a well-defined RA-associated microbiota signature is lacking, so we collected fecal samples from all four groups at five time points (TPs) and determined the dynamic alterations in the microbiome during the development of arthritis by using shotgun metagenomic sequencing. The five TPs included day 0, before AIA induction (TP1), one pre-arthritis time point on day 7 (TP2), and three TPs, day 14 (TP3), day 21 (TP4), and day 30 (TP5), during arthritis progression in the AIA rats (Additional file 5: Figure S5).

**Fig. 2:**
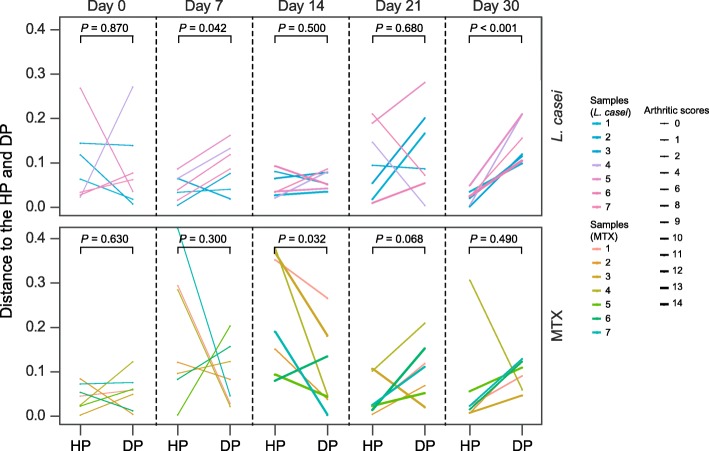
Dynamic changes of the gut microbiota composition in the *L. casei*/MTX-treated rats over time. Distances from healthy plane (HP) and model plane (DP) for each sample of the *L. casei-*/MTX-treated rats on the five time points are shown. The colors of lines correspond to different samples and the thickness reflects the severity of arthritis. Difference between HP and DP is analyzed by paired *t* tests

To determine the dynamics of the changes in the gut microbiome composition in the *L. casei* ATCC334-treated animals, we used a space-based calculation derived from the PCoA of the Bray-Curtis distances of the healthy and vehicle-treated subjects at the species level, which were set as the “healthy plane” (HP) and “disease plane” (DP), respectively. Distance from each sample to the HP and DP revealed dynamic changes in the gut microbiome, and the thickness of the line reflected the severity of arthritis. Samples from the rats treated with *L. casei* exhibited significant closer distances to the HP than the DP at TP2, while for the samples from the MTX-treated rats, there showed an apparent change at TP2 and a significant closer distance to DP than HP at TP3 (Fig. 2), suggesting that *L. casei* might remedy volatile gut dysbiosis at the early stage. At TP5, along with the relief of arthritis observed in *L. casei* group, the composition of the gut microbiomes was significantly closer to HP than DP (Fig. 2), suggesting that dysbiosis in arthritis could be reversed by *L. casei*, which finally contributed to the amelioration of AIA. Interestingly, the arthritic scores were positively correlated with the distance to HP (Spearman’s correlation coefficient = 0.48, *P* = 0.015) and negatively correlated with the distance to DP (Spearman’s correlation coefficient = − 0.46, *P* = 0.019) after adjustment for weight and group, indicating that the alterations in microbiome are closely correlated to the severity of arthritis.

### *L. casei* remedies the perturbed microbiome in AIA rats

To identify the microbial species or strains along with progression and treatment of arthritis, we next conducted a non-negative elastic-net regularized linear regression of the metagenomic sequencing results at the five TPs for each fecal sample against its related arthritis score. In total, 25 microbes were selected, among which *Lachnospiraceae bacterium*, *Proteus mirabilis*, and *Corynebacterium urealyticum* have been found to be related to RA [7, 19]. The relative abundances of the identified 25 microbes were processed by normalization and then presented as log_10_ fold changes at each TPs in comparison to TP 1. In the log_10_ fold change of TP2 to TP1, eight microbes were significantly altered in the model group, among which six microbes were changed in the MTX treated group, and only four were altered in *L. casei* treated rats (Additional file 6: Figure S6), consistent to the result that *L. casei* effectively inhibited RA progression at the very early stage, while at TP3, the alterations of microbes in *L. casei* group were much closer to those in model group (Additional file 7: Figure S7).

**Fig. 3:**
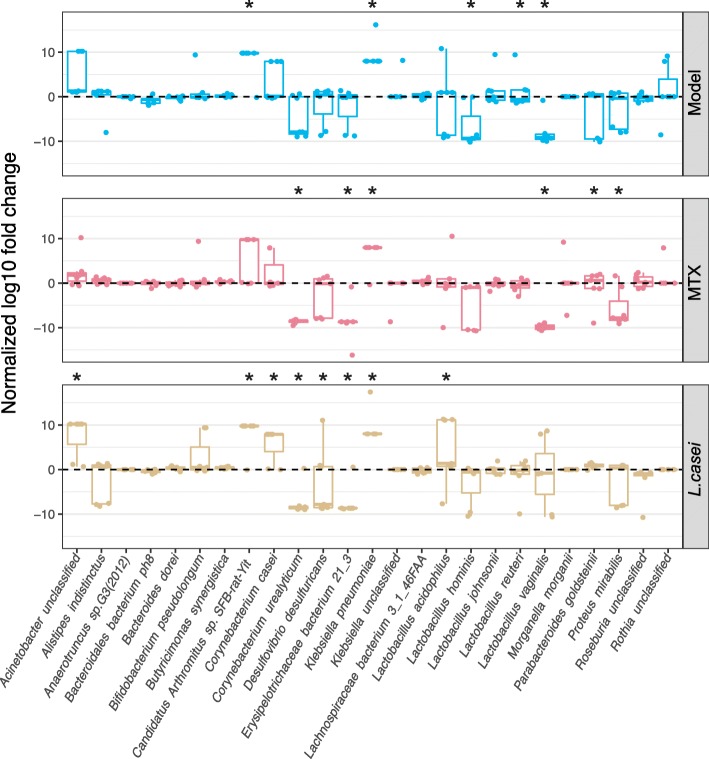
Log_10_ fold change of the relative abundance of arthritis-correlated species at TP5 in comparison with samples of TP1. Boxes represent the median and interquartile ranges (IQRs) between the first and third quartiles; whiskers represent the lowest or highest values within 1.5 times IQR from the first or third quartiles. Circles represent samples. Significant fold change is marked with an asterisk

In the log_10_ fold change of TP5 to TP1, the relative abundances of *Candidatus Arthromitus sp*. *SFB-rat Yit* and *Klebsiella pneumoniae* were increased, while those of *L. hominis*, *L. reuteri*, and *L. vaginalis* were decreased after the induction of arthritis, indicating that these microbes may play important roles in the induction of experimental arthritis (Fig. 3). We further investigated the alterations of arthritis-correlated species in the *L. casei*-treated AIA rats. Unlike rats in the model group, a cluster of microbes including *Acinetobacter* unclassified, *Corynebacterium casei*, and *L. acidophilus* was upregulated (Fig. 3), and *Corynebacterium urealyticum*, *Desulfovibrio desulfuricans*, and *Erysipelotrichaceae* bacterium 21-3 were downregulated*.* Of note, these alterations occurred in the absence of persistent colonization by *L. casei* ATCC334, as we did not detect an enrichment of *L. casei* in the fecal samples isolated from the *L. casei*-treated rats (Additional file 8: Figure S8). However, the relative abundances of other *Lactobacillus* species were upregulated (such as *L. acidophilus*) or restored to the normal level ( such as the downregulated microbes in model group: *L. hominis*, *L. reuteri*, and *L. vaginalis*) (Fig. 3), suggesting that the function of *L. casei* in suppressing arthritis is closely associated with its function in modulating the microbiome, especially other *Lactobacillus* strains.

To determine whether the alterations in the microbiota were specific to *L. casei*, we further compared the differences in microbiome composition between the MTX- and *L. casei*-treated rats. The abundances of *Erysipelotrichaceae* bacterium 21-3, *Corynebacterium urealyticum*, *L. hominis*, and *L. reuteri* were partly regulated by intervention with either of these two treatments (Fig. 3), which indicates that these microbes might be useful as biomarkers of therapeutic responses in RA patients. More interestingly, the abundances of *Desulfovibrio desulfuricans*, *L. acidophilus*, and *L. vaginalis* were effectively modulated by the *L. casei* treatment, but not by the MTX treatment. Thus, *L. casei* ATCC334 is capable of ameliorating arthritis by normalizing the levels of certain affected microbes in AIA rats.

### Inhibition of expression of pro-inflammatory cytokines correlates with the abundance of bacteria affected by administration of *L. casei*.

**Fig. 4:**
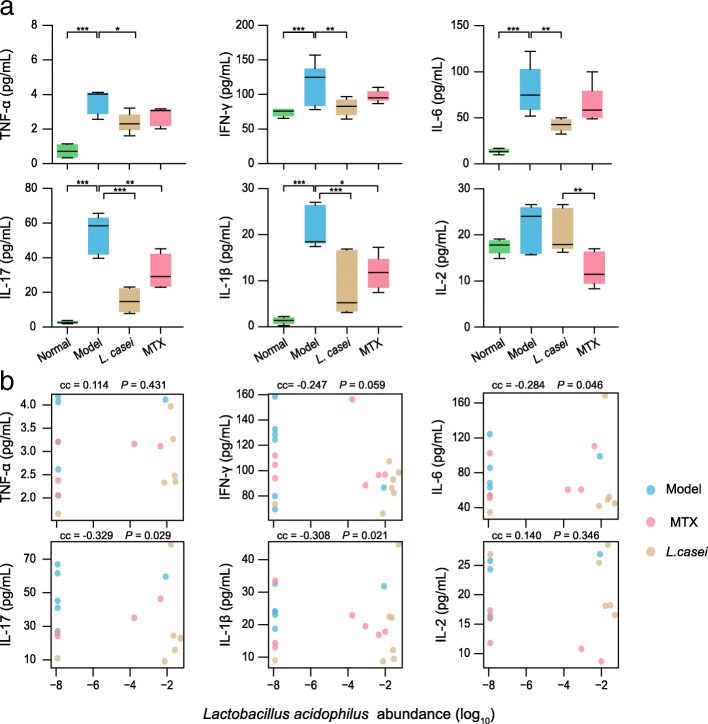
*L. casei* inhibits pro-inflammatory cytokines expression *via* resurrection of *L. acidophilus*. **a** The expressions of cytokines (IL-17, IL-1β, TNFα, IL-6, IFN-γ, IL-2) in serum are assessed using ELISA. Data are shown as mean ± s.e.m and min, max. Differences between groups are analyzed by one-way ANOVA. (*P < 0.05, **P < 0.01, ***P < 0.001 VS model). **b** Associations of the abundance of *L. acidophilus* with plasmatic cytokines. cc, Spearman’s correlation coefficient after adjustment for weight, group, and arthritis score

Cytokines play critical roles in the pathogenesis of RA, while inhibiting expression or function of cytokines increases the probability of remission of RA and protects bones from destruction [20]. Therefore, we determined the serum levels of interferon-γ (IFN-γ), tumor necrosis factor-α (TNF-α), and interleukins (IL)-1β, IL-17, IL-2, and IL-6 and determined the correlations between the levels of pro-inflammatory cytokines and alterations in the gut microbiota after *L. casei* treatment in AIA rats. The results showed a significant reduction in the levels of pro-inflammatory cytokines IFN-γ, TNF-α, IL-1β, IL-17, and IL-6 in the *L. casei*-treated AIA rats compared to the vehicle-treated AIA rats (Fig. 4a). In addition, the levels of IL-17 and IL-1β, which are produced by activated adaptive and innate immune cells [21, 22], respectively, were significantly reduced in the animals treated with *L. casei*. Collectively, these data indicate that the alleviation of arthritis in the *L. casei*-treated rats was related to the suppression of pro-inflammatory cytokine expression.

To better understand the interaction between the abundances of the microbes associated with amelioration of the AIA of rats and the expression levels of cytokines in the *L. casei* group, we computed covariances to analyze whether the relative abundance of *L. casei* or MTX treatment-correlated species (*Erysipelotrichaceae* bacterium 21-3, *Corynebacterium urealyticum*, *Desulfovibrio desulfuricans*, *and Lactobacillus* strains including *L. hominis*, *L. acidophilus*, *L. reuteri*, and *L. vaginalis*) were correlated with the expression of pro-inflammatory cytokines. Results showed that these bacteria were all significantly correlated to one or more inflammatory cytokines (Fig. 4b, Additional file 9: Figure S9, Additional file 10: Figure S10, Additional file 11: Figure S11, Additional file 12: Figure S12, Additional file 13: Figure S13, Additional file 14: Figure S14). Interestingly, most of the rats with enriched *L. acidophilus* in the *L. casei*-treated group showed decreased expression of pro-inflammatory cytokines and significant correlations were detected between the relative abundance of *L. acidophilus* and the expression level of IL-6, IL-17, and IL-1β (*P* < 0.05) (Fig. 4b). These results suggest that the effect of *L. casei* on AIA in rats is associated with the suppressive effect of treatment-correlated microbes on pro-inflammatory cytokine expression.

### Maintenance of the redox balance of oxidative stress is involved in the amelioration of RA in the AIA rats induced by treatment with *L. casei*

**Fig. 5:**
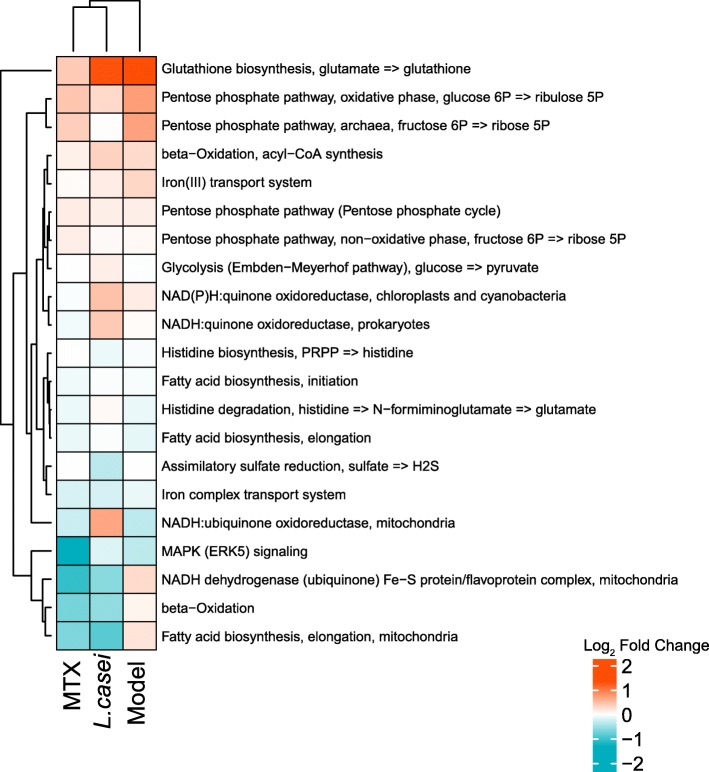
*L. casei* maintains the redox balance of oxidative stress. Mean log_2_ fold changes in the abundance of immune-related KEGG modules at TP5 in comparison with samples of TP1 from MTX, *L. casei*, and model group. The KEGG orthology group modules and groups are ordered by unsupervised hierarchical clustering. Cyan, reduced modules; red, increased modules. Modules missing from one or more groups are not plotted

To explore the potential mechanisms behind the beneficial effects of administration of *L. casei*, we conducted a comprehensive analysis of the functional modules in the gut microbiome of the *L. casei-* and MTX-treated rats using the KEGG database. Consistent with the notion that alterations in the microbiota lead to changes in host metabolism and the redox balance, the functional modules for the pentose phosphate pathway, including the oxidative phase, were found to be enhanced in the AIA rats, which was consistent with the results of previous reports on RA patients [23]. After treatment with *L. casei*, the abundance of genes involved in the pentose phosphate pathway was reduced, and the abundance of genes involved in the nicotinamide adenine dinucleotide (NADH) quinone oxidoreductase, assimilatory sulfate reduction, NADH ubiquinone oxidoreductase, beta-oxidation, and fatty acid biosynthesis modules were increased, suggesting that *L. casei* exerts its anti-arthritic effect through modulating immunometabolism and redox capacity (Fig. 5).

Moreover, *L. acidophilus* has been found to be able to improve the antioxidant status of collagen-induced arthritic rats [24]. These data imply that the modulation of the gut microbiome by *L. casei*, which results in the enrichment of *L. acidophilus*, is able to alleviate arthritis by maintaining the redox balance of oxidative stress.

## Discussion

There is increasing evidence that using intensive DMARD-based strategies in early RA in the clinic is helpful for long-term remission and for achieving positive functional, radiographic, and prognostic outcomes [25, 26]. The concept of a “window of opportunity” was proposed to emphasize the importance of early treatment for RA patients [27], but the strategy focusing on the triggering factors is limited. In the current study, we used a probiotic bacterium, *L. casei*, as a prophylactic treatment for arthritis. Because of its obvious effectiveness and low toxicity, supplementation with *L. casei* is probably an effective option to inhibit RA progression at the very early stage.

Previous studies have found dysbiosis in the gut microbiome of RA patients [5, 7, 28], but little is known about the highly personalized microbiome dynamics during the induction, progression, and treatment of arthritis, especially in regard to the entire genomes of all the organisms present. In the current study, we used shotgun metagenomic surveys and set an HP and DP to summarize the dynamics of all organisms for the first time. The results showed that *L. casei*, regardless of the function of the metabolites that produced by *L. casei*, was capable of maintaining the balance of the microbiome, along with the disease process, at a distance closer to the healthy state than to the disease state of the gut microbiome. These results indicate that *L. casei* could remedy the disordered microbiome caused by the induction of arthritis at the entire genome level, and the beneficial effects induced by the *L. casei* treatment might be attributed to specific alterations in the gut microbiome.

Therapeutic regimens that theoretically target the intestinal flora and the gut-joint axis have been in use since the 1940s [29]. Several regimens, such as sulfasalazine and minocycline [30], have been incorporated into DMARD regimens to treat RA, suggesting that an “optimal” microbiota is able to efficiently optimize immune responses [31]. In addition, probiotics have been shown to facilitate the efficacy of anticancer immunotherapies, such as anti-PD-L1 therapy, an immune checkpoint blockade approach [32, 33]. A previous study also showed that probiotics, such as *L. casei* 01, might help alleviate the symptoms of RA and suppress pro-inflammatory cytokines in patients undergoing treatment with DMARDs [10, 34], suggesting that there is a positive synergistic effect between DMARDs and probiotics on arthritis, which was also confirmed in our study. Our current study showed that a significant reduction in the levels of pro-inflammatory cytokines was detected after 30 days of prophylactic treatment with *L. casei*. Though no enrichment of *L. casei* was detected in the fecal samples isolated from the *L. casei*-treated rats, the relative abundance of *L. acidophilus*, *L. hominis*, *L. reuteri*, and *L. vaginalis* were increased by the administration of *L. casei* to the AIA rats, indicating that *L. casei* ameliorates arthritis mainly through rebalancing the *Lactobacillus* strains. *Lactobacillus* are the most prominent probiotics that are often present in the gastrointestinal tract. Some strains of *Lactobacillus* have been demonstrated to drive T cell differentiation from intraepithelial CD4+ T cells into immunoregulatory T (Treg) cells by generating indole derivatives of tryptophan that activate the aryl-hydrocarbon receptor of CD4+ T cells [35]. The metabolic products of these *Lactobacillus* strains, such as short-chain fatty acids, also promote the regulation of colonic Treg cell homeostasis [36, 37]. However, there remains to be confirmed that the difference of *L. casei*-triggered alterations of the microbiome in gastrointestinal tissues are the same to that in the fecal samples as is reported before [38], so as to draw a conclusion that *L. casei* exerts its anti-arthritis effect via modulation of *Lactobacillus* strains.

In addition, we found that *L. casei* could markedly increase the *L. acidophilus* population in the gut microbiota of the AIA rats, which is in line with previous reports on *L. acidophilus* [39, 40]. Although the mechanism by which *L. acidophilus* modulates T helper (Th) 17 cell differentiation and production in the gut remains unclear, the downregulation of signal transducer and activator of transcription (STAT) 3 transcription [41] is one of the main molecular mechanisms by which *L. acidophilus* inhibits the IL-23/Th17 axis. Furthermore, *L. acidophilus* was confirmed to inhibit bone loss and increase bone heterogeneity in osteoporotic mice, suggesting that the protective effect of *L. casei* on the bones of AIA rats might partly be attributed to the modulation of *L. acidophilus.*

Reactive oxygen species (ROS) were initially regarded as merely damaging agents but now are recognized as second messengers that regulate cellular function through oxidant signaling [42]. The targeting of ROS generation is developing into a potential therapeutic approach for RA patients [43]. Naïve T cells from RA patients demonstrated that the replenishment of intracellular ROS corrected abnormal proliferative behavior in T cells and successfully suppressed synovial inflammation [23]. Modulating the redox balance and gut microbiome at the entire genome level might be involved in the anti-arthritic effect of *L. casei* on AIA rats [44]. The restored microbiome modulated by *L. casei* suppressed the activated pentose phosphate pathway and improved the NADH quinone oxidoreductase, assimilatory sulfate reduction, NADH ubiquinone oxidoreductase, beta-oxidation, and fatty acid biosynthesis modules. Lactobacilli show a distinct capacity for inducing cellular ROS generation [45]. Moreover, intestinal epithelial cells that came into contact with lactobacilli exhibited increased protein oxidation, resulting in the induction of the transcription of redox-stimulated modules, such as the nuclear factor (erythroid-derived 2)-like 2 (Nrf2) pathway [46]. Thus, restoring oxidant signaling via the modulation of the gut microbiome by *L. casei* may suppress arthritis through the suppression of pro-arthritogenic T cell effector functions.

The effects of *L. casei* in the occurrence, development, and treatment of arthritis are still not fully understood. We anticipate that large-scale studies will be focused on the potential roles of gut microbiome on RA, especially for the early diagnosis and early treatment of disease.

## Conclusions

This current work is the first to identify the compositional and functional alterations in the gut microbiome by using a time-series research protocol in AIA rats, and the results support the conclusion that modulating the microbiota could be a new strategy for the prevention and treatment of human RA. Of note, these results were obtained in an experimental arthritis rat model, and there is a strong need to perform intensive investigations in RA patients. The precise molecular mechanisms behind the preventive/therapeutic actions of probiotics must be further elucidated, and the identified bacterial markers in the gut need to be intensively validated.

## Additional files

Additional file 1: **Figure S1.** The differences of optical density in the concentration of *L. casei* pretreated by CMC-Na or MRS. (PDF 17 kb)

Additional file 2: **Figure S2.** The growth states of CMC-Na or MRS pretreated *L. casei* after culturing for 48 h in MRS agar. (PDF 29 kb)

Additional file 3: **Figure S3.** The arthritic scores in AIA rats. (PDF 54 kb)

Additional file 4: **Figure S4.** The alterations of trabecular bone mineral density (BMD), bone volume rate (BV/TV), trabecular number (Tb.N), porosity percent (Po × total) and tissue mineral density (TMD) tested by Micro CT. (PDF 41 kb)

Additional file 5: **Figure S5.** The source of fecal samples for assessment the alterations of gut microbiome in AIA rats. (PDF 412 kb)

Additional file 6: **Figure S6.** The Log_10_ fold change of the relative abundance of arthritis-correlated species at TP2 in comparison with samples of TP1. (PDF 205 kb)

Additional file 7: **Figure S7.** The Log_10_ fold change of the relative abundance of arthritis-correlated species at TP3 in comparison with samples of TP1. (PDF 206 kb)

Additional file 8: **Figure S8.** Abundance of *L. casei* among fecal samples of different group. (PDF 1030 kb)

Additional file 9: **Figure S9.** Associations of the abundance of *D. desulfuricans* with plasmatic cytokines. (PDF 147 kb)

Additional file 10: **Figure S10.** Associations of the abundance of *Erysipelotrichaceae bacterium 21_3* with plasmatic cytokines. (PDF 149 kb)

Additional file 11: **Figure S11.** Associations of the abundance of *Corynebacterium urealyticum* with plasmatic cytokines. (PDF 209 kb)

Additional file 12: **Figure S12.** Associations of the abundance of *L. hominis* with plasmatic cytokines. (PDF 148 kb)

Additional file 13: **Figure S13.** Associations of the abundance of *L. reuteri* with plasmatic cytokines. (PDF 142 kb)

Additional file 14: **Figure S14.** Associations of the abundance of *L. vaginalis* with plasmatic cytokines. (PDF 145 kb)

Additional file 15: **Table S1.** Metagenomic sequencing data for the SD rat microbiome. (XLSX 19 kb)

Additional file 16: **Table S2.** Relative abundances of species determined by the metagenomic sequencing. (XLSX 35 kb)

## Data Availability

Metagenomic sequencing data for all samples have been deposited in the European Nucleotide Archive (ENA) database under the accession identification number PRJEB22973. Other relevant data have been deposited in the GigaScience Database (GigaDB, http://gigadb.org/dataset/100440).

## Abbreviations

AIA: Adjuvant-induced arthritis
BMD: Trabecular bone mineral density
BV/TV: Bone volume rate
CFA: Complete Freund’s adjuvant
CFU: Colony-forming units
CMC-Na: Sodium carboxymethyl cellulose
*D*. *desulfuricans*: *Desulfovibrio desulfuricans*
DMARDs: Disease-modifying anti-rheumatic drugs
DP: Disease plane
ELISA: Enzyme-linked immunosorbent assay
ENA: European nucleotide archive
ESR: Erythrocyte sedimentation rate
HP: Healthy plane
ICSH: International Council for Standardization in Haematology
IFN-γ: Interferon-γ
IL: Interleukin
KEGG: Kyoto Encyclopedia of Genes and Genomes
*L*. *casei*: *Lactobacillus casei*
MHC: Major histocompatibility complex
micro-CT: Micro-computed tomography
MTX: Methotrexate
NADH: Nicotinamide adenine dinucleotide
Nrf2: Nuclear factor (erythroid-derived 2)-like 2
PCoA: Principal coordinates analysis
Po × total: Porosity percent
RA: Rheumatoid arthritis
ROS: Reactive oxygen species
SD: Sprague-Dawley
SE: Single-end
STAT: Signal transducer and activator of transcription
Tb.N: Trabecular number
Th: T helper
TMD: Tissue mineral density
TNF-α: Tumor necrosis factor-α
TP: Time point
Treg: Regulatory T
