# Supplementary figures and images for: A single bacterium restores the microbiome dysbiosis to protect bones from destruction in a rat model of rheumatoid arthritis
Source: Microbiome. 2019 Jul 17;7:107. doi: 10.1186/s40168-019-0719-1 (PMC6637628; doi:10.1186/s40168-019-0719-1)

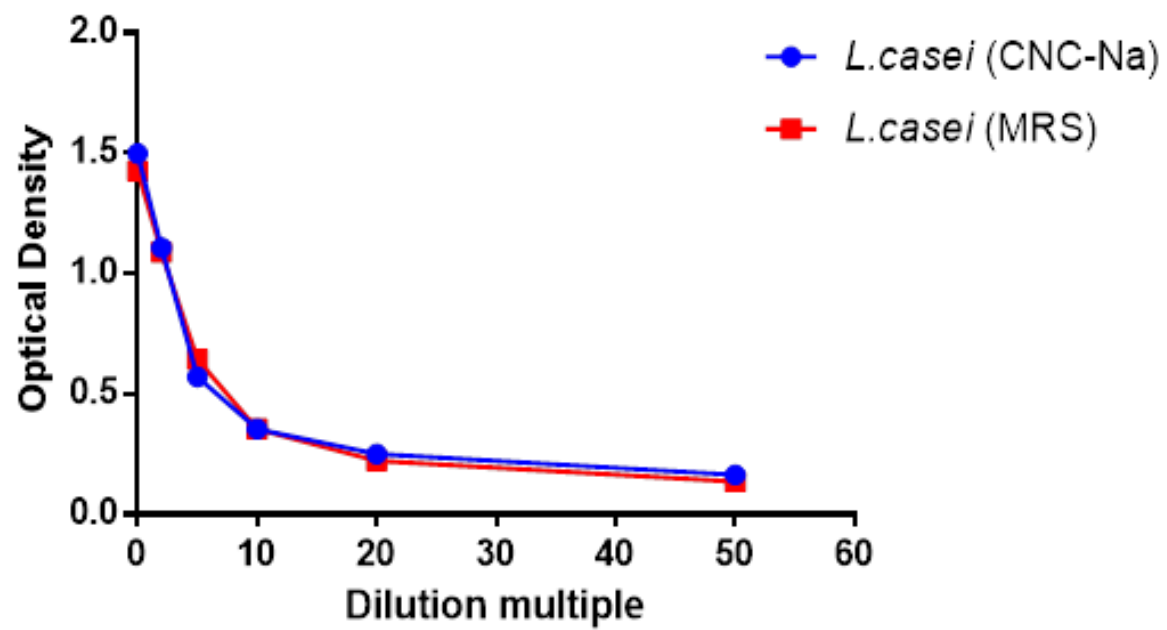

Supplement: Supplementary file 1 — Figure S1. The differences of optical density in the concentration of L. casei pretreated by CMC-Na or MRS. (PDF 17 kb) [file 40168_2019_719_MOESM1_ESM.pdf]

Vehicle Control

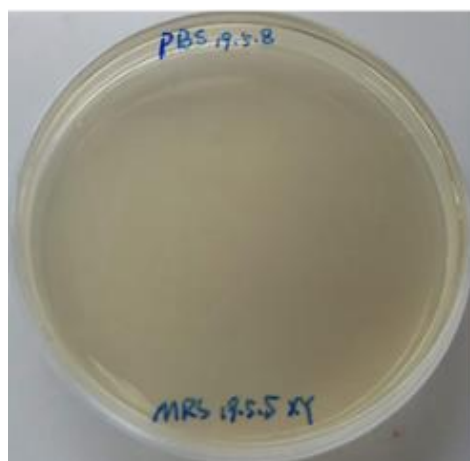

*L.casei*(MRS)

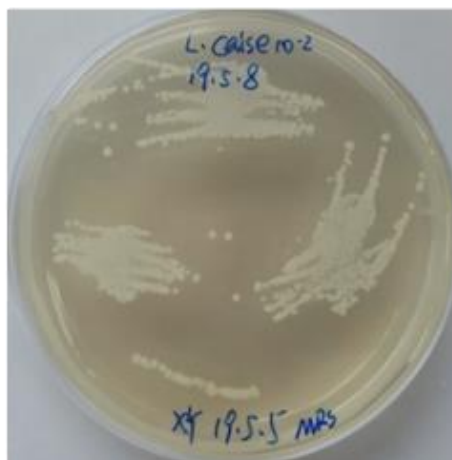

*L.casei* (CNC-Na)

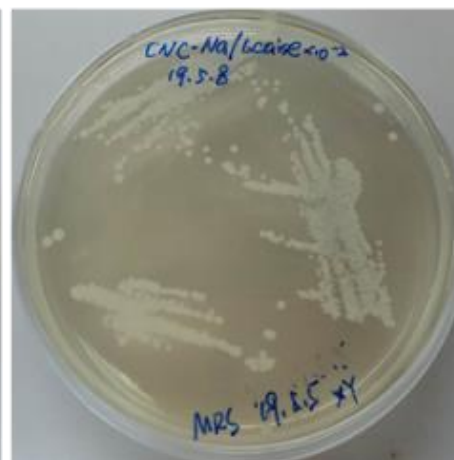

Supplement: Supplementary file 2 — Figure S2. The growth states of CMC-Na or MRS pretreated L. casei after culturing for 48 h in MRS agar. (PDF 29 kb) [file 40168_2019_719_MOESM2_ESM.pdf]

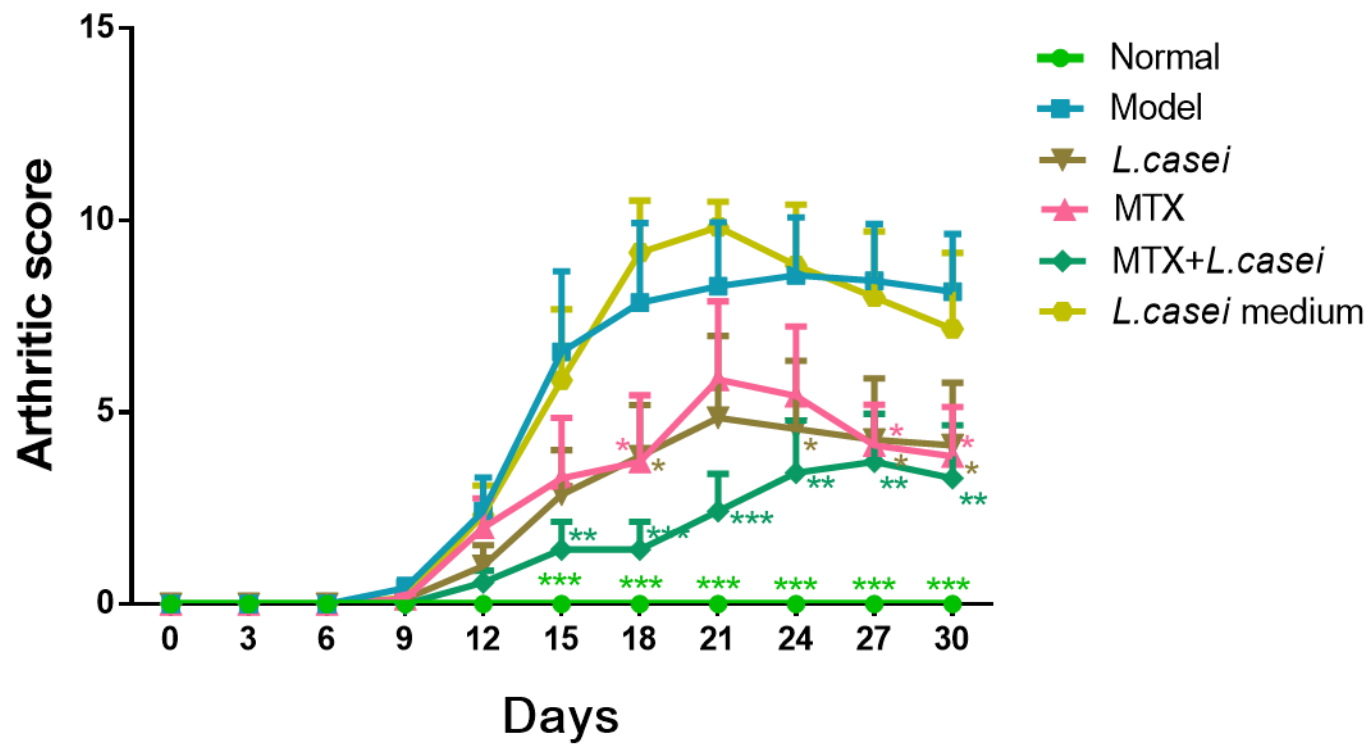

Supplement: Supplementary file 3 — Figure S3. The arthritic scores in AIA rats. (PDF 54 kb) [file 40168_2019_719_MOESM3_ESM.pdf]

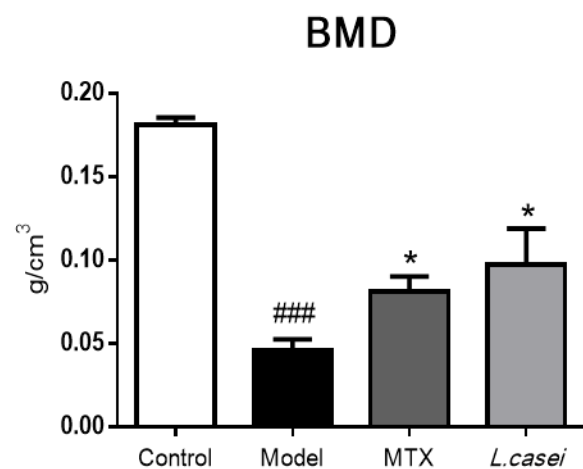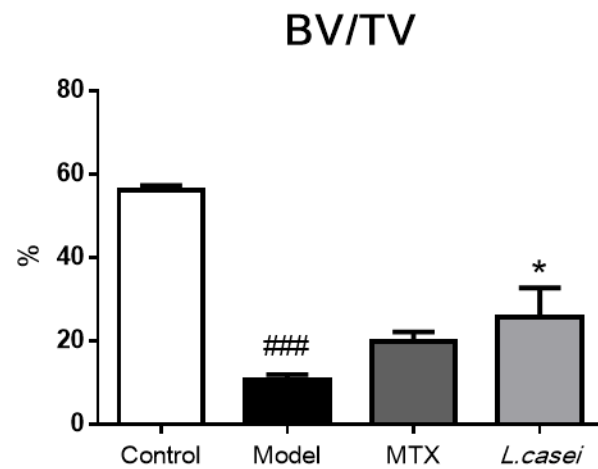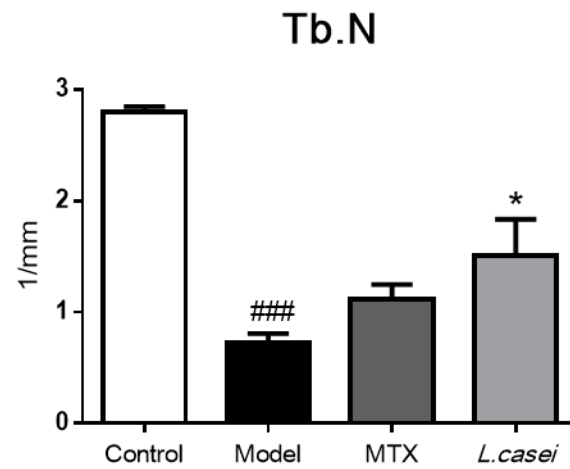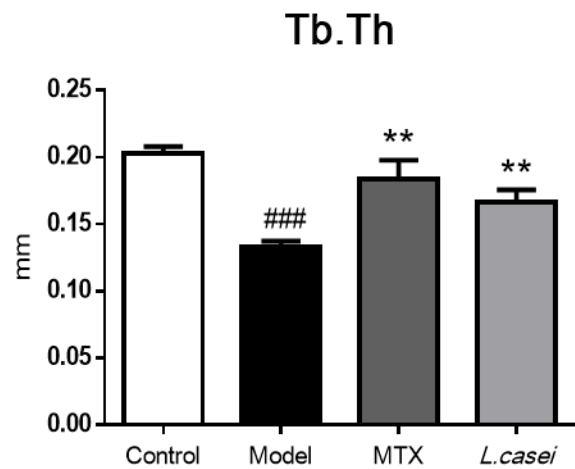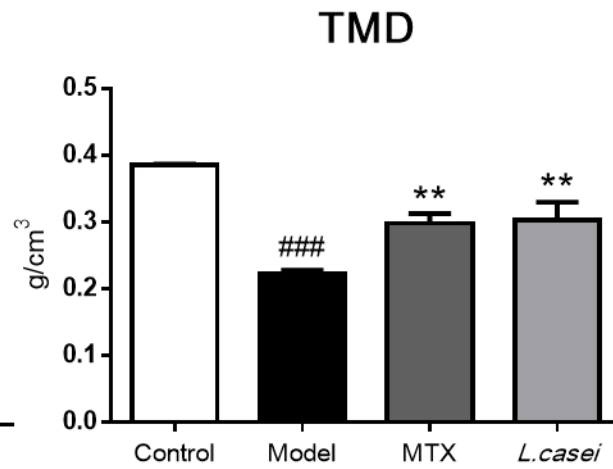

Supplement: Supplementary file 4 — Figure S4. The alterations of trabecular bone mineral density (BMD), bone volume rate (BV/TV), trabecular number (Tb.N), porosity percent (Po × total) and tissue mineral density (TMD) tested by Micro CT. (PDF 41 kb) [file 40168_2019_719_MOESM4_ESM.pdf]

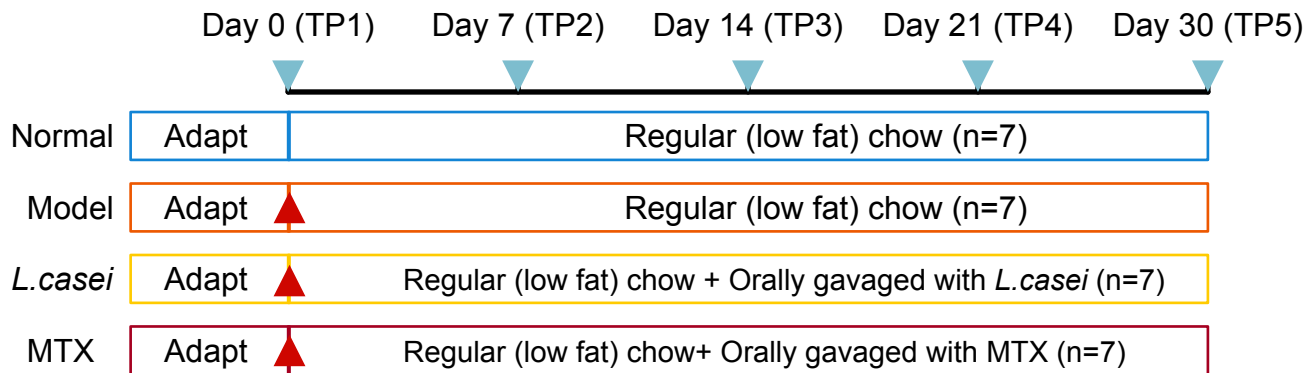

▼ Collection of feces

▲ CFA induction of arthritis

TP: Time point

Supplement: Supplementary file 5 — Figure S5. The source of fecal samples for assessment the alterations of gut microbiome in AIA rats. (PDF 412 kb) [file 40168_2019_719_MOESM5_ESM.pdf]

Normalized log10 fold change

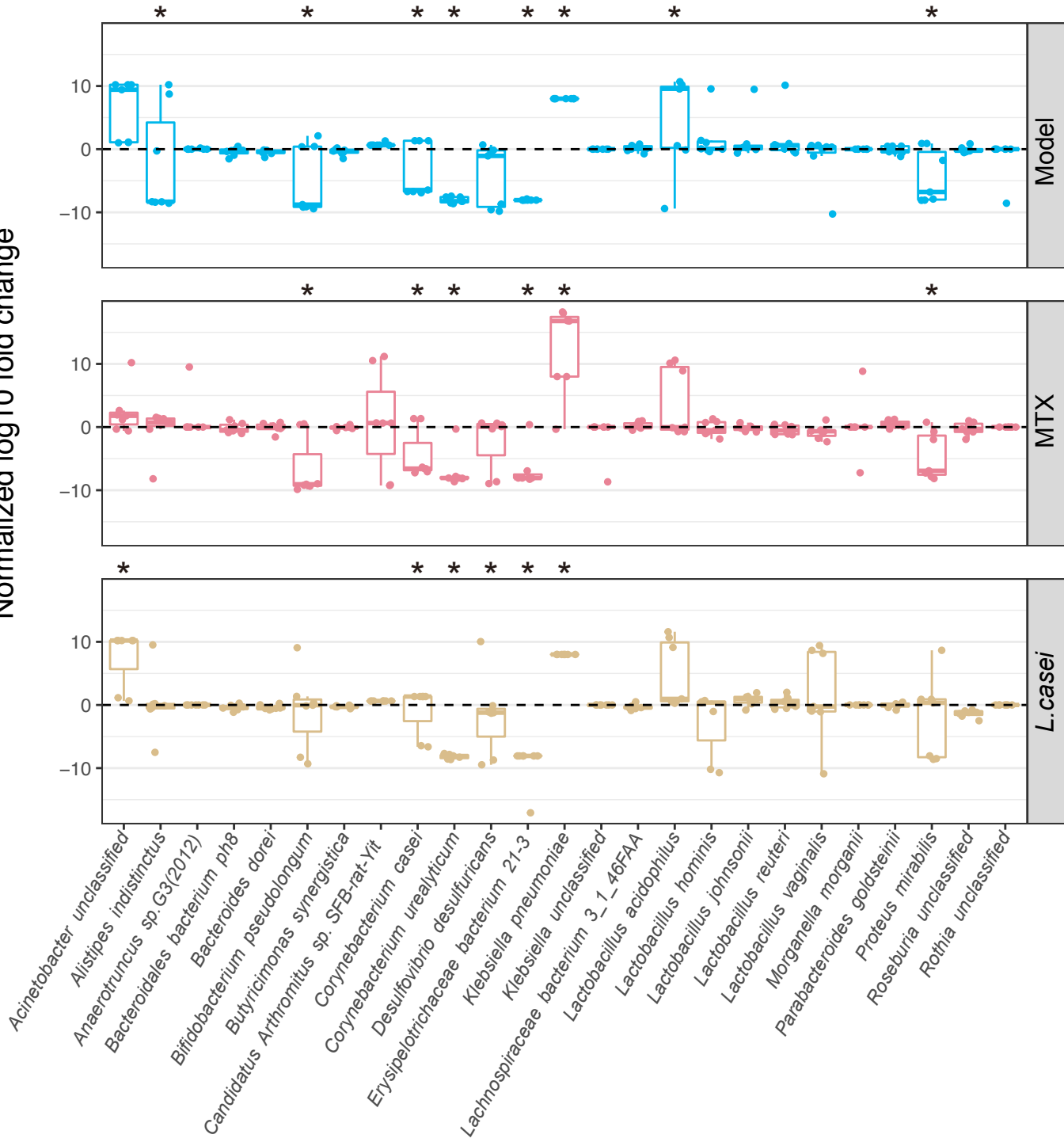

Supplement: Supplementary file 6 — Figure S6. The Log10 fold change of the relative abundance of arthritis-correlated species at TP2 in comparison with samples of TP1. (PDF 205 kb) [file 40168_2019_719_MOESM6_ESM.pdf]

Normalized log10 fold change

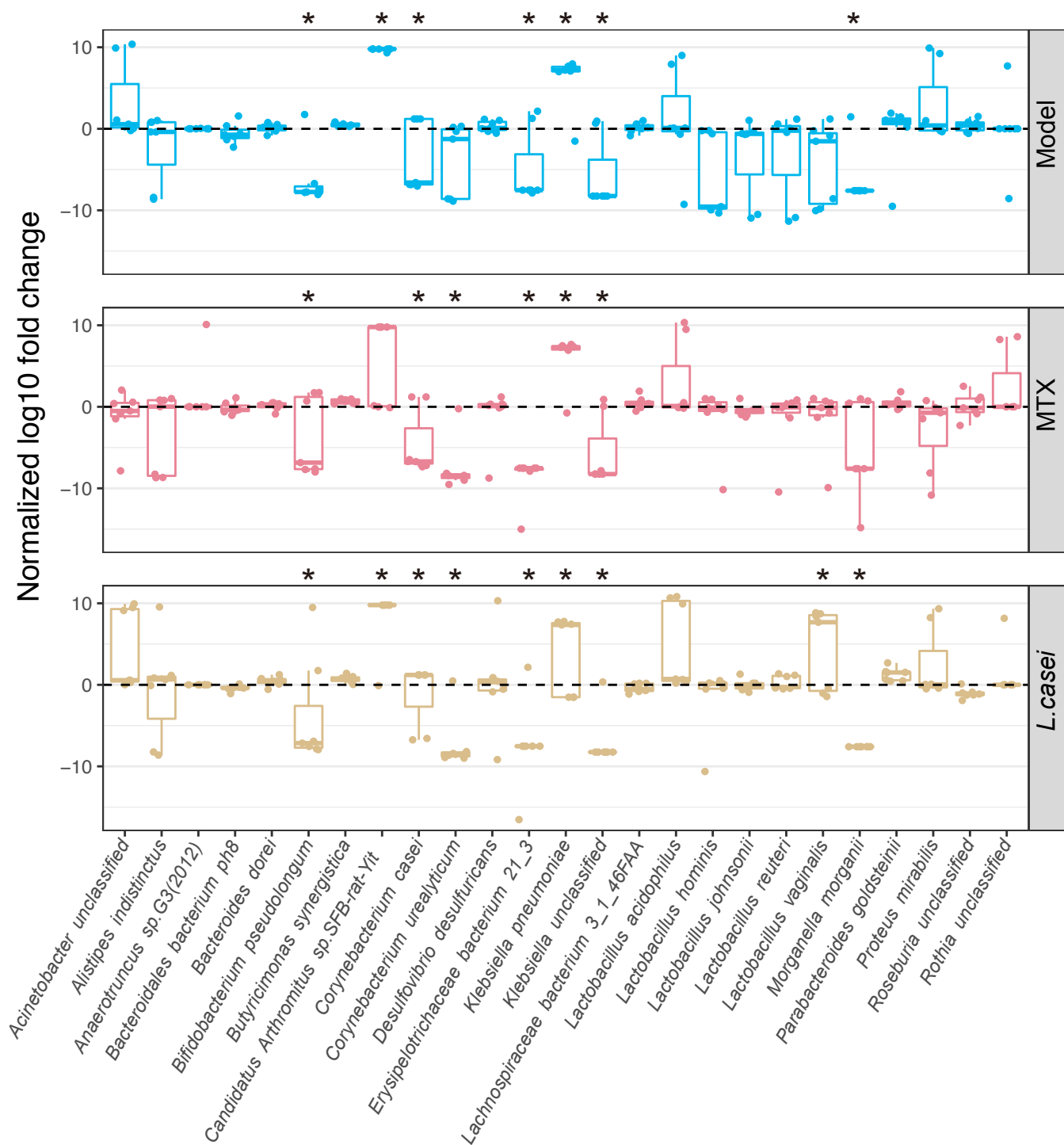

Supplement: Supplementary file 7 — Figure S7. The Log10 fold change of the relative abundance of arthritis-correlated species at TP3 in comparison with samples of TP1. (PDF 206 kb) [file 40168_2019_719_MOESM7_ESM.pdf]

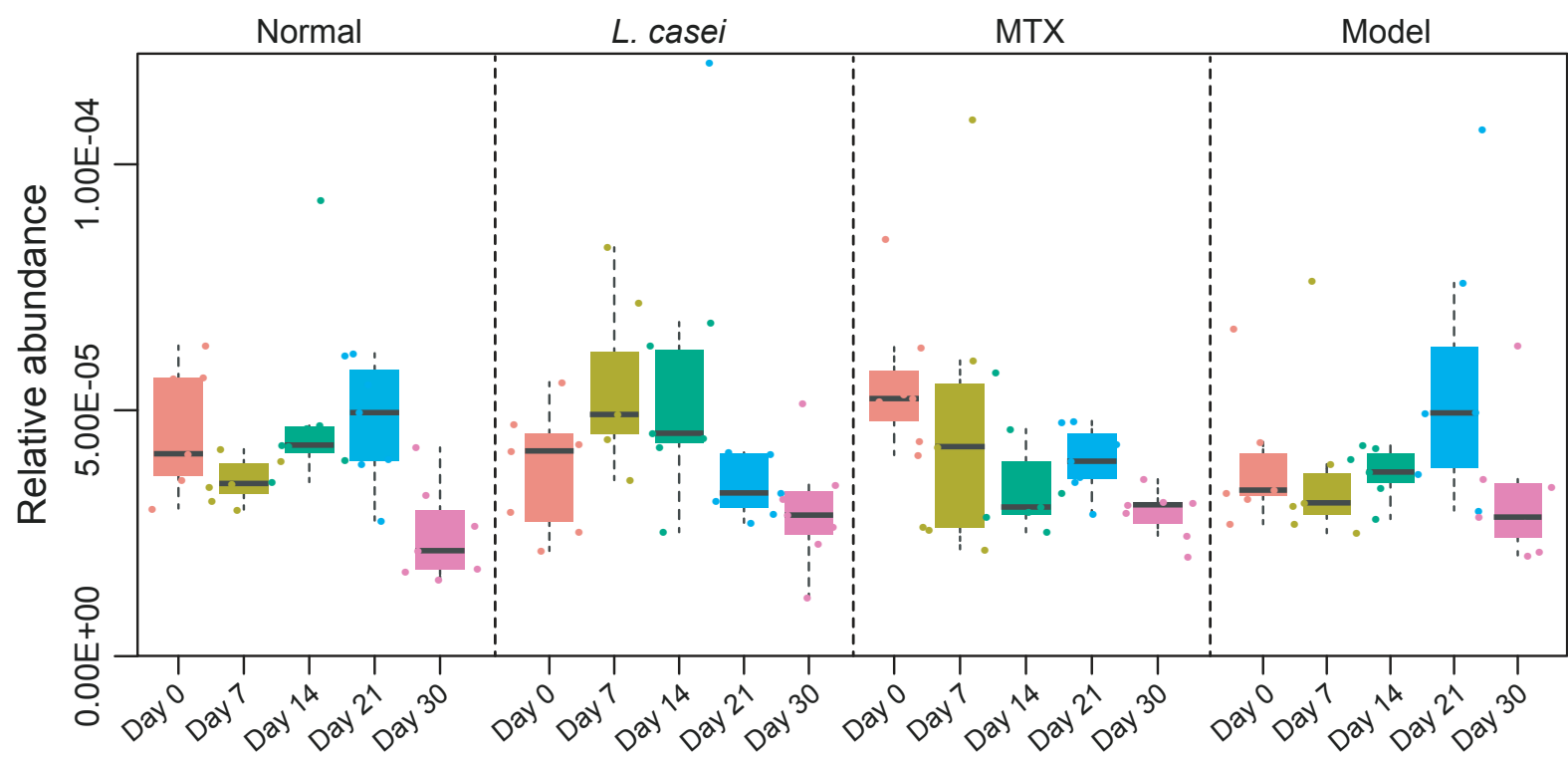

Supplement: Supplementary file 8 — Figure S8. Abundance of L. casei among fecal samples of different group. (PDF 1030 kb) [file 40168_2019_719_MOESM8_ESM.pdf]

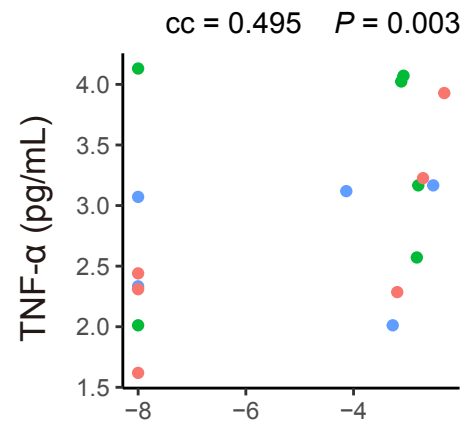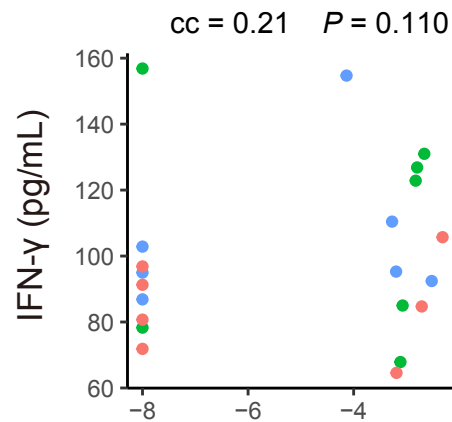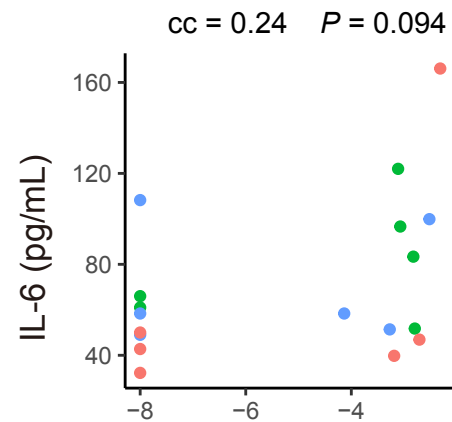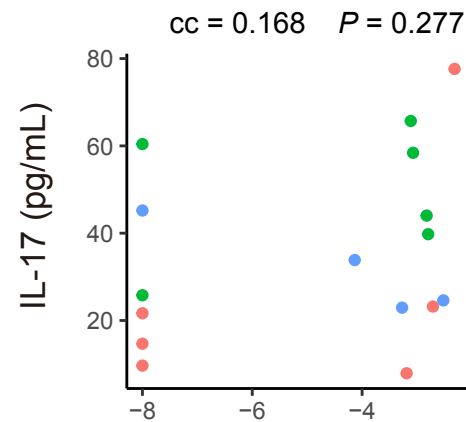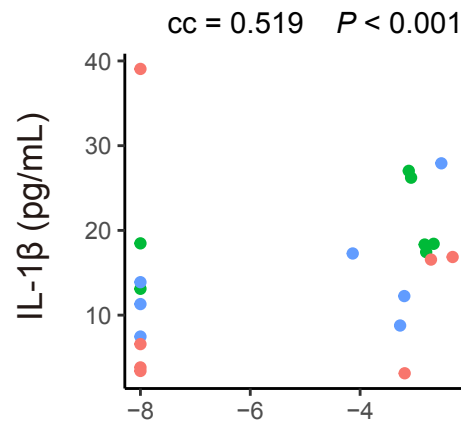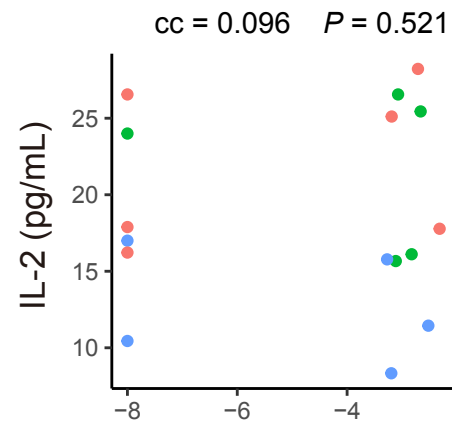

● *L.casei*  
● Model  
● MTX

*Desulfovibrio desulfuricans* abundance ( $\log_{10}$ )

Supplement: Supplementary file 9 — Figure S9. Associations of the abundance of D. desulfuricans with plasmatic cytokines. (PDF 147 kb) [file 40168_2019_719_MOESM9_ESM.pdf]

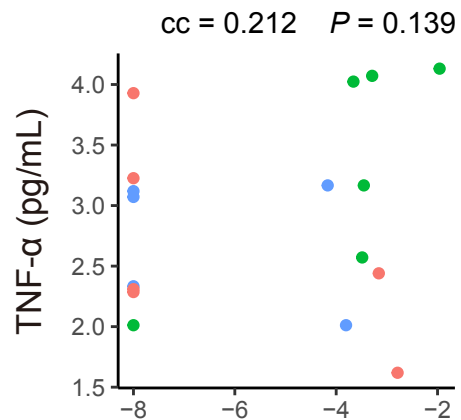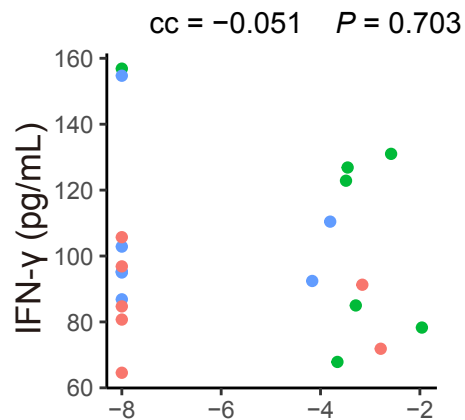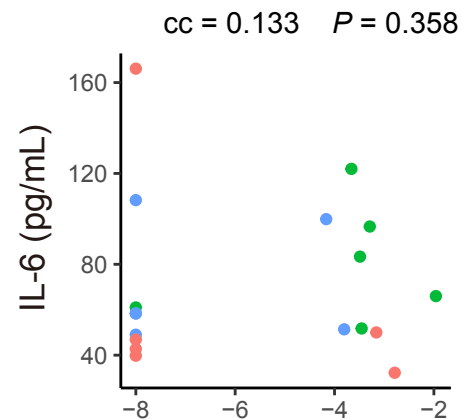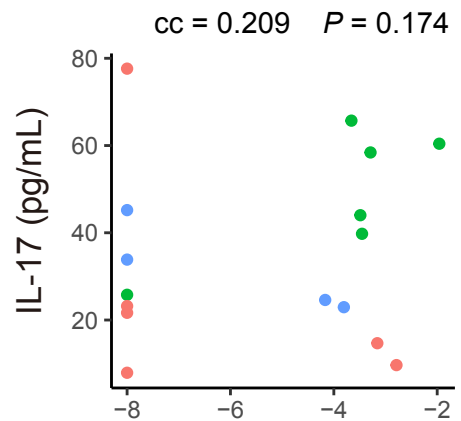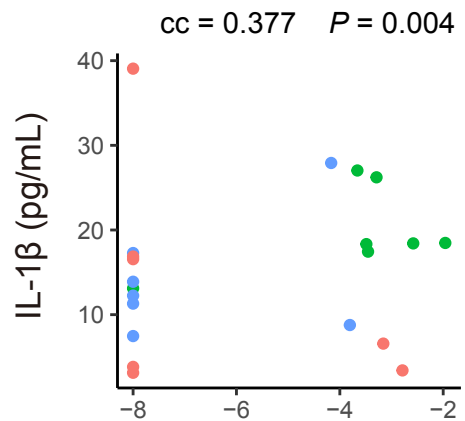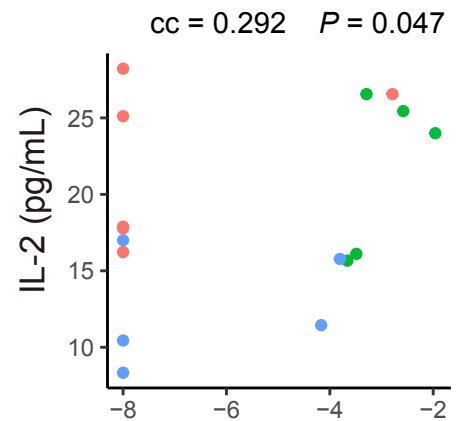

*Erysipelotrichaceae bacterium 21\_3* abundance ( $\log_{10}$ )

Supplement: Supplementary file 10 — Figure S10. Associations of the abundance of Erysipelotrichaceae bacterium 21_3 with plasmatic cytokines. (PDF 149 kb) [file 40168_2019_719_MOESM10_ESM.pdf]

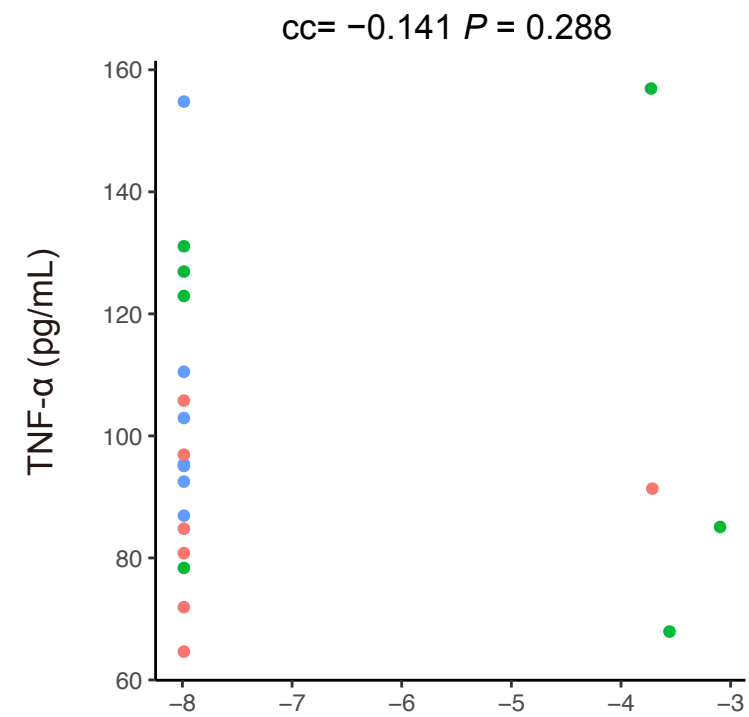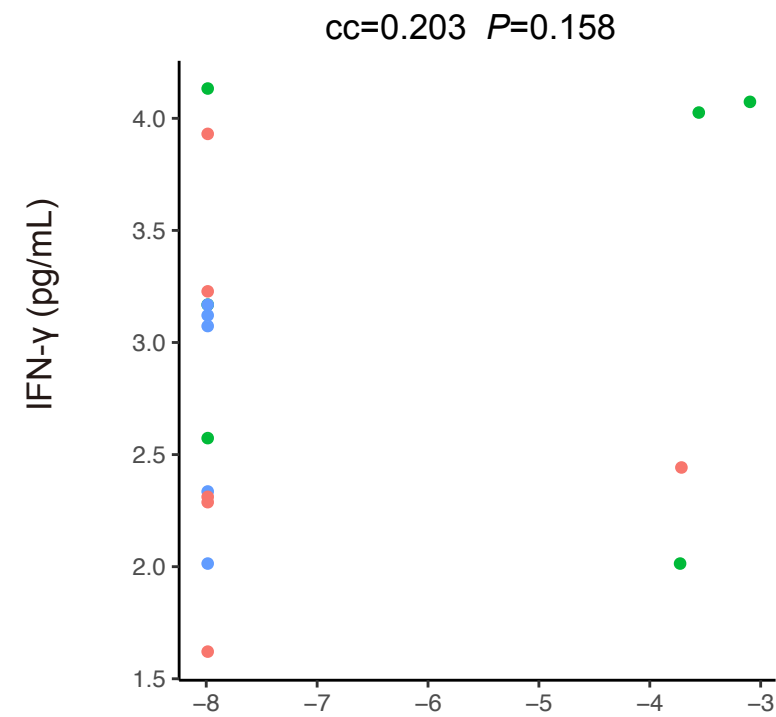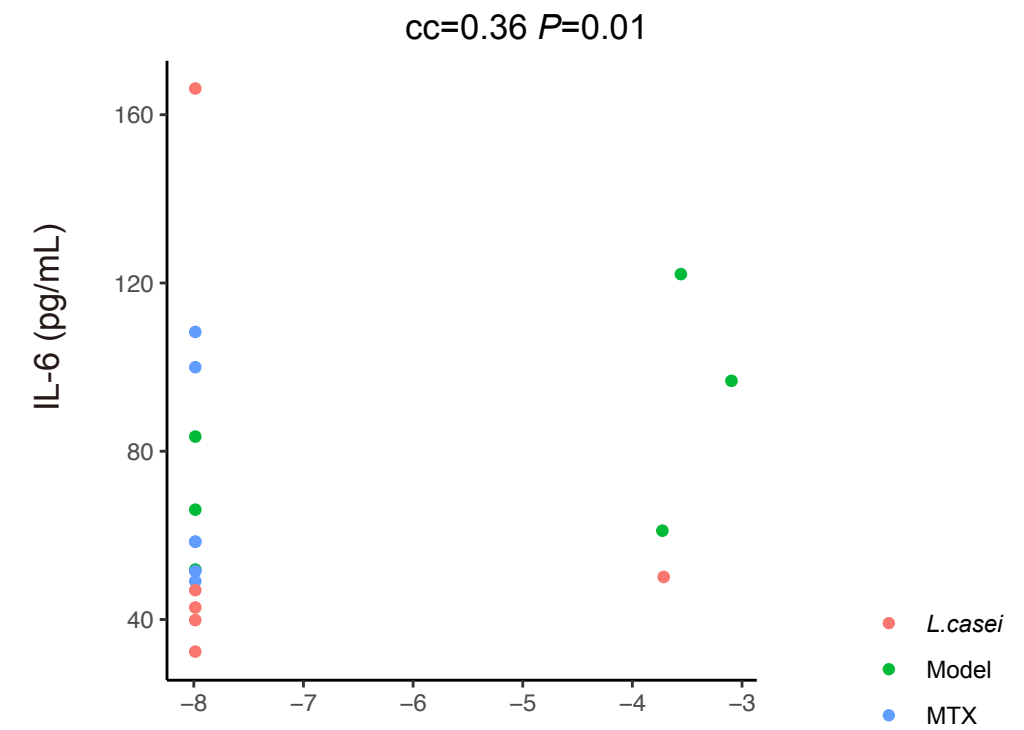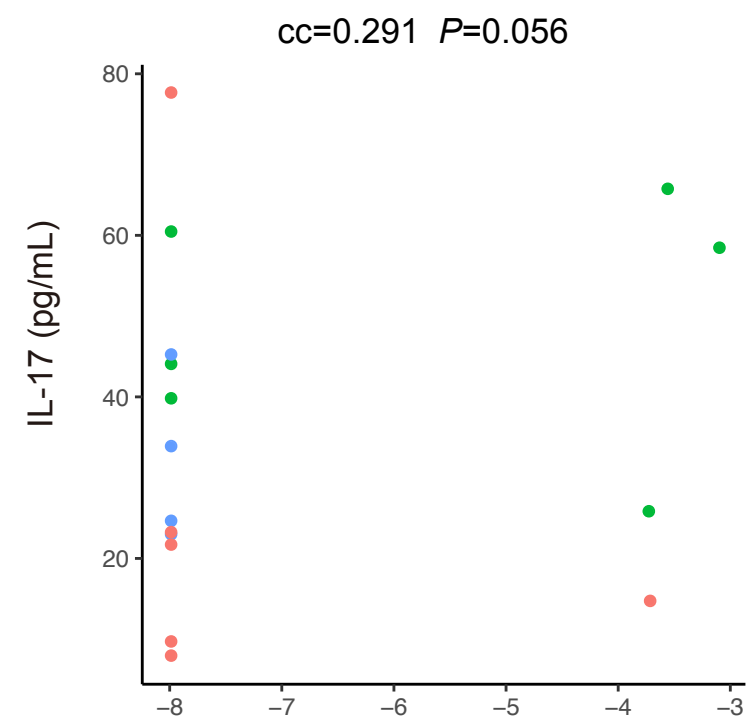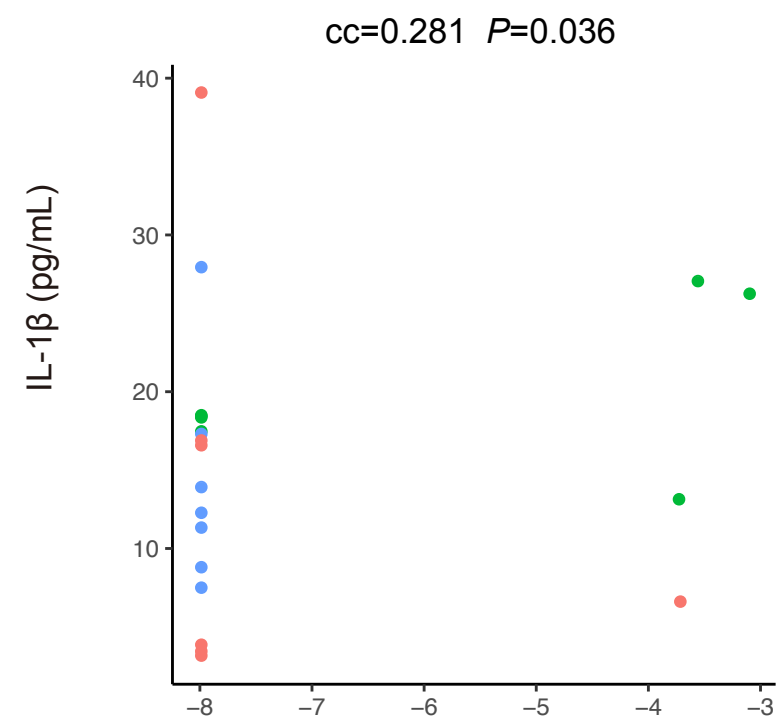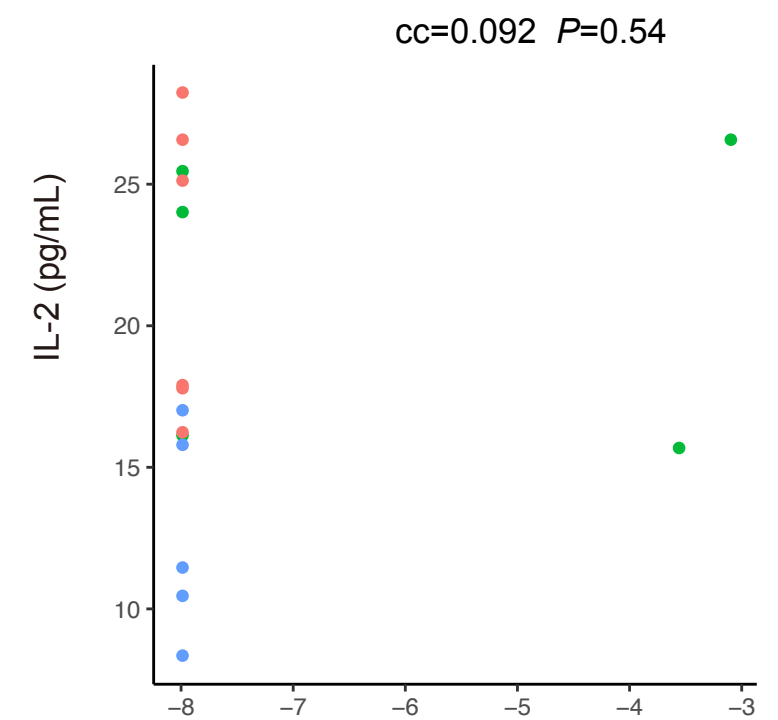

*Corynebacterium urealyticum* abundance ( $\log_{10}$ )

Supplement: Supplementary file 11 — Figure S11. Associations of the abundance of Corynebacterium urealyticum with plasmatic cytokines. (PDF 209 kb) [file 40168_2019_719_MOESM11_ESM.pdf]

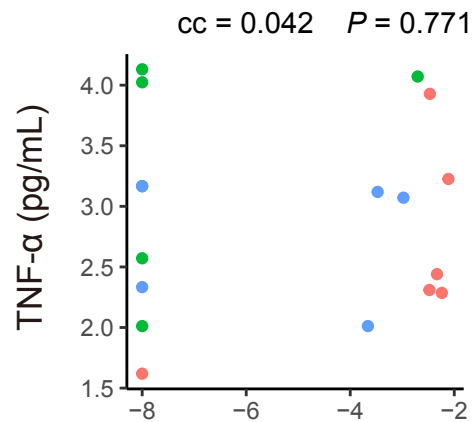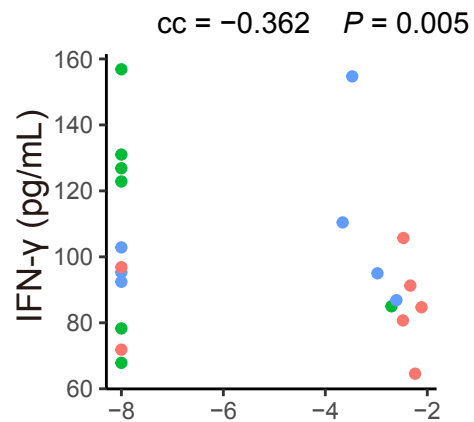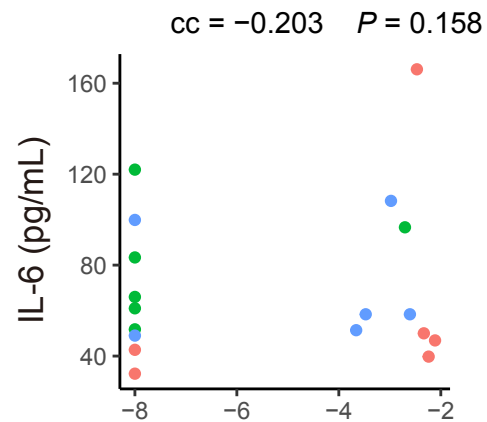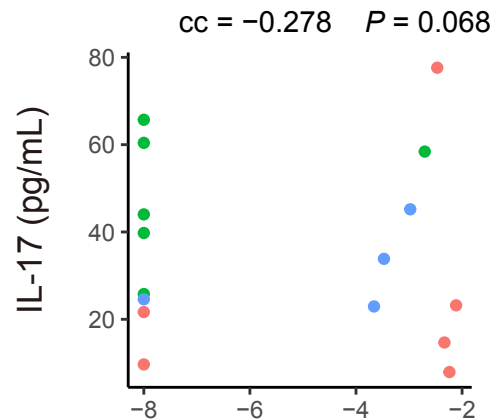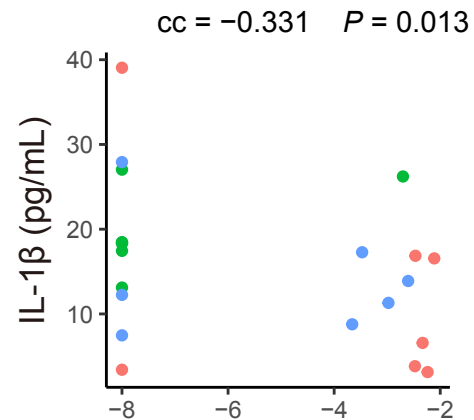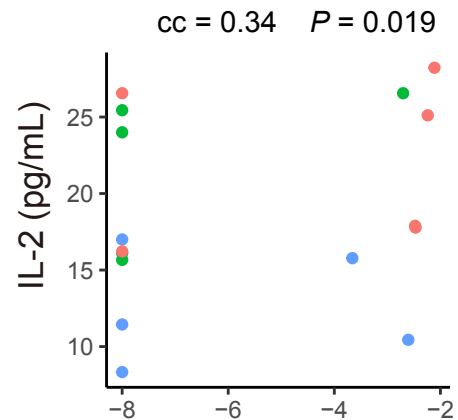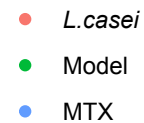

*Lactobacillus hominis* abundance (log<sub>10</sub>)

Supplement: Supplementary file 12 — Figure S12. Associations of the abundance of L. hominis with plasmatic cytokines. (PDF 148 kb) [file 40168_2019_719_MOESM12_ESM.pdf]

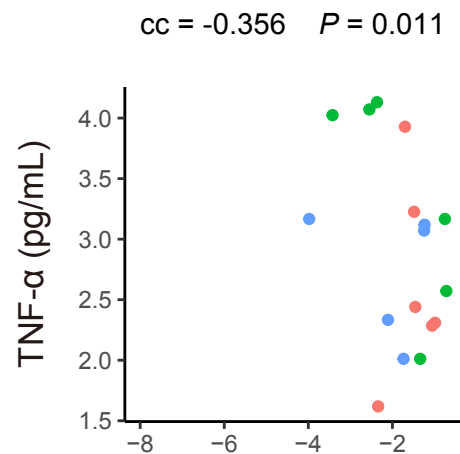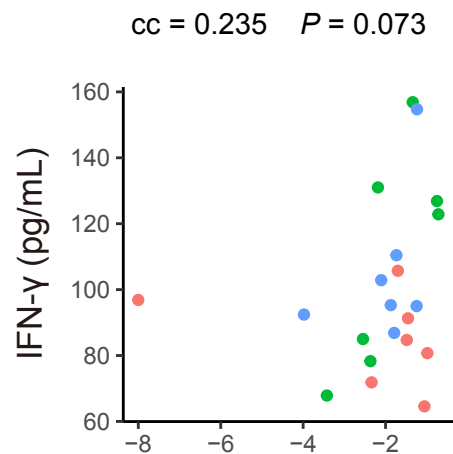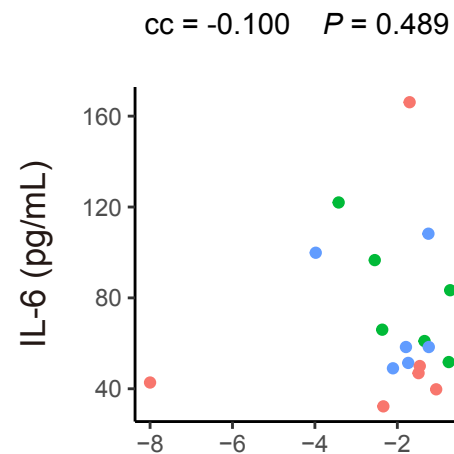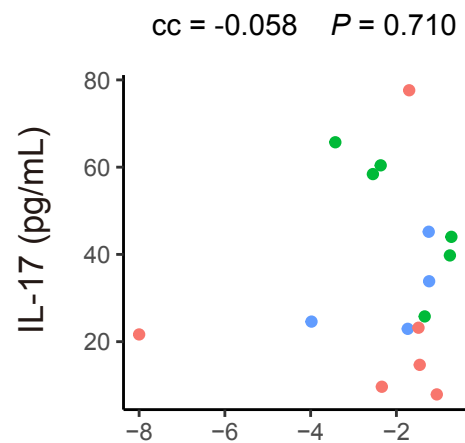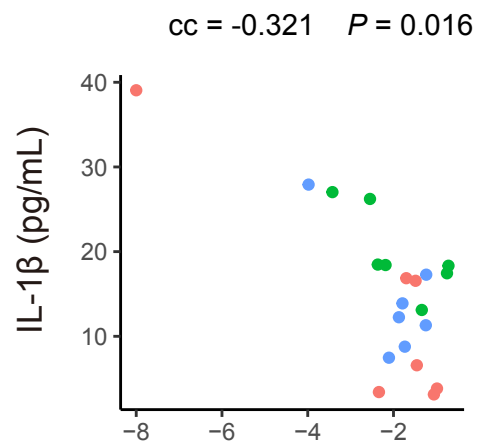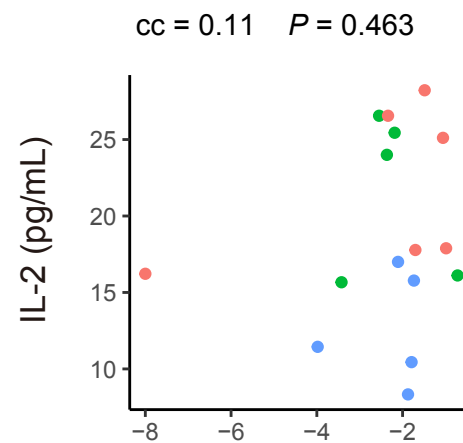

*Lactobacillus reuteri* abundance ( $\log_{10}$ )

Supplement: Supplementary file 13 — Figure S13. Associations of the abundance of L. reuteri with plasmatic cytokines. (PDF 142 kb) [file 40168_2019_719_MOESM13_ESM.pdf]

cc = -0.183  $P = 0.202$

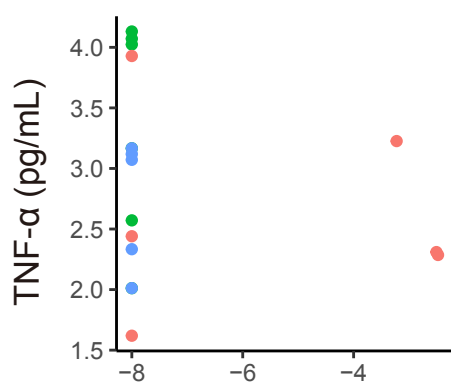

cc = -0.475  $P = 0.001$

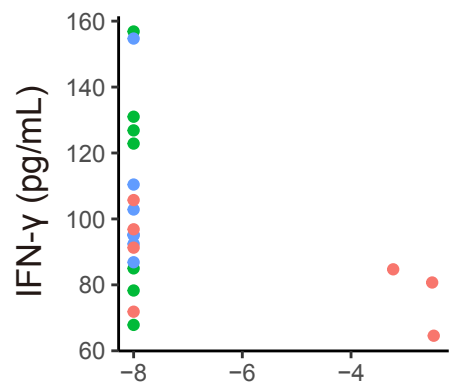

cc = -0.436  $P = 0.002$

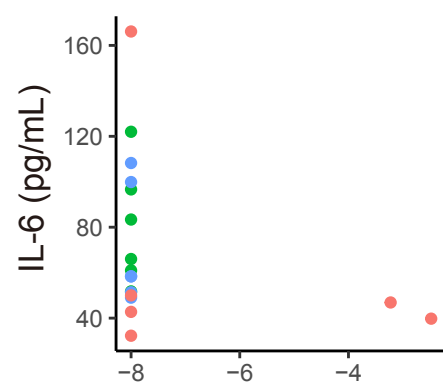

*L.casei*  
Model  
MTX

cc = -0.407  $P = 0.006$

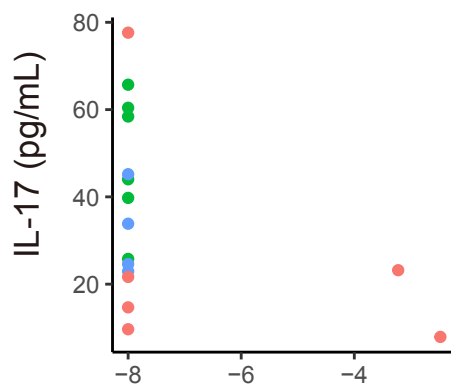

cc = -0.414  $P = 0.002$

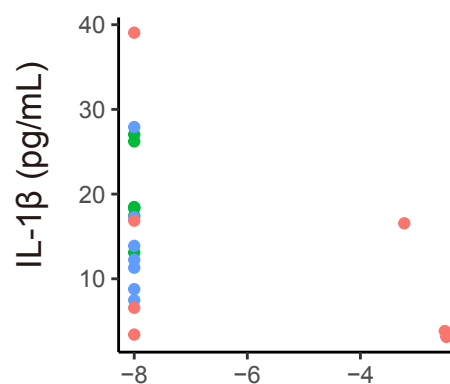

cc = 0.406  $P = 0.005$

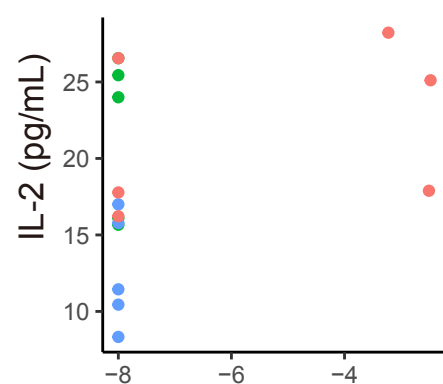

*Lactobacillus vaginalis* abundance (log<sub>10</sub>)

Supplement: Supplementary file 14 — Figure S14. Associations of the abundance of L. vaginalis with plasmatic cytokines. (PDF 145 kb) [file 40168_2019_719_MOESM14_ESM.pdf]
